# Impact of High-Pressure Homogenization on the Cell Integrity of *Tetradesmus obliquus* and Seed Germination

**DOI:** 10.3390/molecules27072275

**Published:** 2022-03-31

**Authors:** Alice Ferreira, Daniel Figueiredo, Francisca Ferreira, Belina Ribeiro, Alberto Reis, Teresa Lopes da Silva, Luisa Gouveia

**Affiliations:** 1 LNEG—UBB—National Laboratory of Energy and Geology I.P., Bioenergy and Biorefineries Unit, Estrada do Paço do Lumiar 22, 1649-038 Lisbon, Portugal; alice.ferreira@lneg.pt (A.F.); francisca.opf@gmail.com (F.F.); belina.ribeiro@lneg.pt (B.R.); alberto.reis@lneg.pt (A.R.); teresa.lopessilva@lneg.pt (T.L.d.S.); 2 GreenCoLab—Green Ocean Technologies and Products Collaborative Laboratory, CCMAR, Algarve University, 8005-139 Faro, Portugal; danielfigueiredo@greencolab.com

**Keywords:** microalgae, swine wastewater, flow cytometry, biofertiliser, biostimulant

## Abstract

Microalgae have almost unlimited applications due to their versatility and robustness to grow in different environmental conditions, their biodiversity and variety of valuable bioactive compounds. Wastewater can be used as a low-cost and readily available medium for microalgae, while the latter removes the pollutants to produce clean water. Nevertheless, since the most valuable metabolites are mainly located inside the microalga cell, their release implies rupturing the cell wall. In this study, *Tetradesmus obliquus* grown in 5% piggery effluent was disrupted using high-pressure homogenization (HPH). Effects of HPH pressure (100, 300, and 600 bar) and cycles (1, 2 and 3) were tested on the membrane integrity and evaluated using flow cytometry and microscopy. In addition, wheat seed germination trials were carried out using the biomass at different conditions. Increased HPH pressure or number of cycles led to more cell disruption (75% at 600 bar and 3 cycles). However, the highest increase in wheat germination and growth (40–45%) was observed at the lowest pressure (100 bar), where only 46% of the microalga cells were permeabilised, but not disrupted. Non-treated *T. obliquus* cultures also revealed an enhancing effect on root and shoot length (up to 40%). The filtrate of the initial culture also promoted shoot development compared to water (21%), reinforcing the full use of all the process fractions. Thus, piggery wastewater can be used to produce microalgae biomass, and mild HPH conditions can promote cell permeabilization to release sufficient amounts of bioactive compounds with the ability to enhance plant germination and growth, converting an economic and environmental concern into environmentally sustainable applications.

## 1. Introduction

Global demographic pressure on agricultural production has been overwhelming on non-renewable inputs of fertilisers and pesticides, with drastic consequences to human health and the environment. Every year, 100 million tonnes of synthetic fertilisers are used globally, being responsible for greenhouse gas emissions, on-site soil degradation [1], infertility and biodiversity losses [2], water contamination (ocean dead zones, eutrophication), and human diseases [3]. At the same time, livestock, namely pig farming, generates wastewater at rates of 4 to 8 L per pig per day [4]. This piggery wastewater (PWW) is a very complex effluent, rich in organic matter, ammonia, and suspended solids, which is usually left in decompose in stationary ponds, releasing problematic greenhouse gases such as CO_2_ and N_2_O [5]. Efficient technologies to treat PWW and that are integrated in a circular approach, for instance by generating sustainable agricultural added value products, are necessary to mitigate the impact of this industry. This is especially important since some organic fertilisers, such as animal manure, biosolids from human wastes, anaerobic digestate, biochar or crop residues [6] have associated ecological risks related to veterinary antibiotics [7] or high metal contents, such as Cu or Zn in pig feed [8] or arsenic [9]. Alternative sources of organic fertilisers that improve soil structure and provide plants with a balanced mix of macro and micronutrients will be highly beneficial for the agribusiness sector, while a more efficient and less polluting livestock industry is key for a more sustainable world.

Microalgae have been attracting the interest of farmers and agrochemical industries due to their composition in bioactive compounds, such as phytohormones [10], protein, carbohydrates [11], amino acids [12], polysaccharides [13], or minerals [14]. These have biostimulant and biofertiliser properties, which enhance nutrient use efficiency, rooting, crop yield, quality, and resilience to environmental stresses [15,16]. Moreover, capturing nutrient runoff from wastewaters using microalgae could also counter the eutrophication of natural water bodies [17], while reducing microalgae production costs. Hence, enclosing a more sustainable method for “closed-loop” nutrient cycling and the use of biomass for fertilisers/stimulants are key for an authentic circular economy.

The biochemical composition of microalgae should be as much as possible explored in a biorefinery platform to take advantage of all interesting fractions towards zero waste. The most interesting and abundant microalgae compounds (e.g., protein, sugars, lipids, etc.) are intracellular, which makes cell wall disruption a mandatory process. Several disruption methodologies have been applied, such as enzymatic treatments, microwave-assisted processes, pulsed electric fields (PEF) or high-pressure homogenization (HPH). Carullo et al. (2018) [18] demonstrated that it was possible to selectively recover small-sized cytoplasmic compounds from *Chlorella vulgaris* using PEF, and high molecular weight intracellular components using HPH. Furthermore, Zhang et al. (2019) [19] and Carullo et al. (2022) [20] showed a higher potential of this technique to release water-soluble proteins. HPH is highly efficient in rupturing the strong microalgae cell wall, easy to scale up and optimize (as disruption efficiency is related to a few operation variables, such as pressure or microalgae species) [21], and can be used in aqueous environments avoiding the need of a previous energy intensive drying step. High-pressure homogenizers consist of a chamber where high pressure is applied to force the liquid sample contained therein through a valve or membrane with very narrow slits. This act causes high shear, a large pressure drop and cavitation, all acting to homogenize the sample.

It is still debatable the need for cell disruption to exploit the benefits of microalgae as biofertilisers/biostimulants since some works such as Navarro-López et al. (2020) [22] and Ferreira et al. (2021) [23] showed increments on the germination index (GI) with microalgae added to seeds without any pre-treatment. Figueiredo et al. (2022) [24] also showed the possibility of using mineral enriched whole-cell microalgal biomass to fulfil plants’ nutrient requirements in replacement of chemical sources. Moreover, Stirk et al. (2020) [25] claimed that freeze-drying was sufficient to release the active compounds using water extracts on mung bean while mentioning that sonication and ball-milling had negative impacts on biostimulant results. Navarro-López et al. (2020) [26] also observed that lower HPH pressures induced a better biostimulant effect on germination and plant development. This suggests that permeabilised but not fully disrupted cells are sufficient to release the bioactive compounds. Nevertheless, further studies are necessary to evaluate in more depth the degree of cell disruption and biostimulant activities, which to the best of the authors knowledge are still lacking in the current literature.

Several methods have traditionally been used to track and quantify cell rupture, such as observation under optical or fluorescence microscopy. However, these methods are time and labour demanding and inaccurate, leading to misestimation. Flow cytometry (FC) is a laser-based technology that allows the simultaneous analysis of the chemical and physical characteristics of particles in a fluid. It can measure the optical and fluorescence characteristics of a single cell or any other particle [27]. It is a fast and useful technique to monitor cell physiological status at line near real time contrarily to conventional analytical techniques [28]. FC also allows to monitor and decide the best cultivating strategies to induce the production of a desired metabolite, the best time to harvest cells or to follow different microbial populations in symbiosis [29]. Another use of FC is to determine microalgae cell viability [30]. Cell death is often associated with none or reduced enzymatic activity but can also be associated with impaired or loss of membrane integrity. For cell viability assessment, the most common technique is the fluorescence-stained nuclei, which uses membrane-impermeable fluorochromes (e.g., SYTOX Green, PICO Green) to determine cell viability in terms of membrane integrity [31,32]. Intact cell membranes can be visualized as red-coloured cells, which are a result of autofluorescence, whereas SYTOX permeabilised membranes are observed by the green fluorescence colour.

*Tetradesmus obliquus* was previously shown to successfully treat PWW, with consequently better biomass growth, while promoting the germination of various seeds [23]. However, this microalga possesses a very robust cell wall that requires harsher methods, such as HPH, to break the cells and facilitate the release of the intracellular compounds. In this work, HPH was used to disrupt *T. obliquus* cells grown in 5% piggery wastewater. The treatment performance was assessed by FC coupled with SYTOX Green to provide more depth on cellular membrane integrity, distinguishing between disrupted, permeabilised and intact cell populations. Furthermore, different disruption pressures (100, 350 and 600 bar for 1 cycle) were tested to evaluate how the compromising of the microalga cell wall (permeabilised or disrupted), and thus the potentially higher availability of bioactive compounds, would affect the germination of wheat seeds and further root and shoot growth.

## 2. Results and Discussion

### 2.1. Evaluation of High-Pressure Homogenization (HPH)

#### 2.1.1. Flow Cytometry

**Figure 1:**
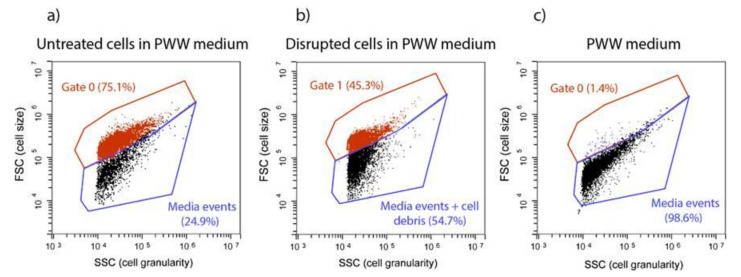
Flow cytometry populations gates present in (**a**) untreated cells in PWW media, (**b**) disrupted cells in PWW media using FSC/SSC dot plots, (**c**) piggery wastewater (PWW) media.

Flow cytometry (FC) analysis detected changes in *T. obliquus* cells after HPH treatment (Figure 1). Figure 1a concerns the density plot of the untreated sample. Events displayed in Gate 0 shows the untreated microalgae cells that maintained their size and structure. On the other hand, events in Gate 1 (Figure 1b) found at lower Forward (FSC) and Side Scatter (SSC) signals, correspond to cells that reduced their size and lost their structure and internal content, as a result of high shear stress.

The piggery wastewater (PWW) used as cultivation media contained particles (as noise), at a low percentage (1.4%; Figure 1c), which appeared in Gate 0, the same gate of *T. obliquus* untreated cells. Therefore, running previously the PWW without cells is necessary to subtract these particles to the total events displayed in Gate 0, to obtain accurate *T. obliquus* untreated cells number (Figure 1a,c).

**Figure 2:**
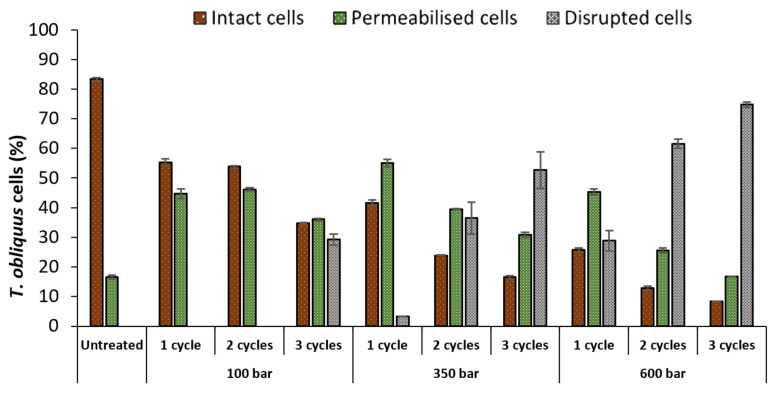
Percentages of intact, permeabilised and disrupted *Tetradesmus obliquus* cells after high-pressure homogenization at different pressure or cycles, determined by flow cytometry. Data are shown as mean ± standard deviation (*n* = 2).

The untreated culture contained 83% of intact cells and 17% of permeabilised cells (Figure 2). HPH treatments affected *T. obliquus* depending on pressure or number of cycles. As expected, the percentage of intact cells decreased, as the pressure or the number of cycles increased. For instance, using 100 bar at 1 and 2 cycles, was already enough to reduce the percentage of intact cells (83 to 54%) and increase the permeabilised population (17 to 46%), though not sufficient to induce cell disruption until cycle 3, attaining 29%. Only after 350 bar and 2 cycles, the percentage of disrupted *T. obliquus* cells surpassed the intact cells (>26%), reaching a maximum of 75% at 600 bar and 3 cycles.

Additionally, the number of permeabilised cells decreased as the pressure or cycle number increased. Indeed at 350 bar, the permeabilised cells proportion decreased from 55 to 40%, and then to 31%, after 1, 2 and 3 cycles, respectively. This decrease was accompanied by the increase in the proportion of disrupted cells (3 to 37%, then to 53%, after 1, 2 and 3 cycles, respectively).

The increase in pressure decreased the number of intact cells, but the maximum tested pressure (600 bar) at 1 cycle achieved only partial disruption (disrupted cells = 29%). Therefore, multiple cycles combined with higher pressure are necessary to achieve a complete cell disruption.

**Figure 3:**
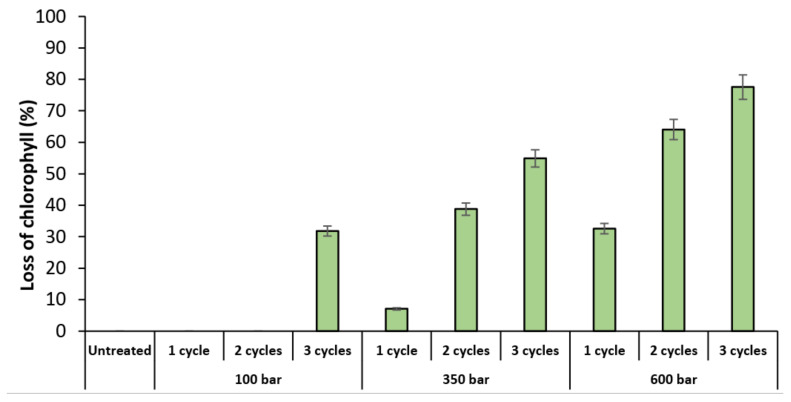
Percentage of *Tetradesmus obliquus* chlorophyll lost as a result of high-pressure homogenization at different pressure or cycles, determined by flow cytometry. Data are shown as mean ± standard deviation (*n* = 2).

Another method to evaluate the impact of the HPH treatment is the detection of lost cell chlorophyll content, which can be seen in the PC5.5 channel. Figure 3 shows the percentage of *T. obliquus* cells that lost chlorophyll content after the HPH treatment. The profile is quite similar to the profile of the disrupted cells (Figure 2), which means that these cells were exposed to such mechanical forces that lost their content, or their chlorophyll pigment was degraded.

#### 2.1.2. Microscopy

**Figure 4:**
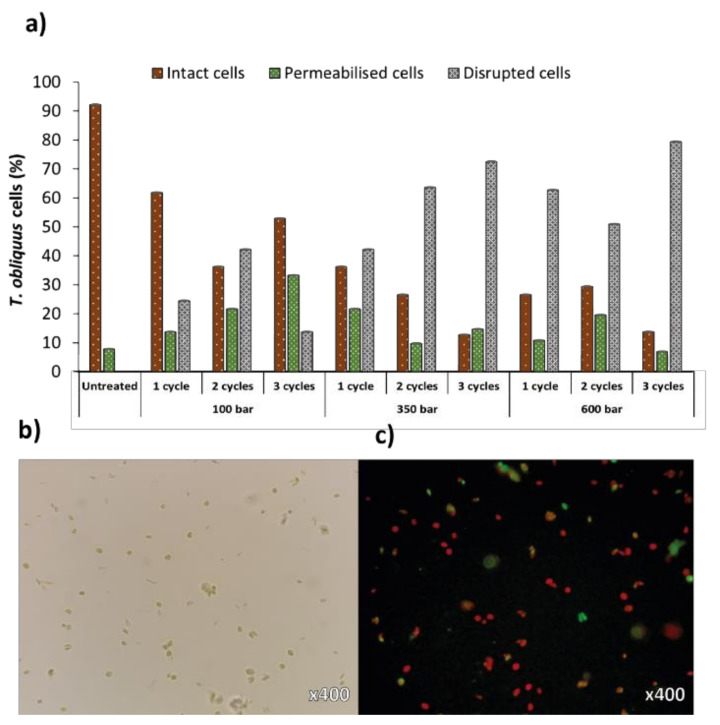
(**a**) Percentages of intact, permeabilised and disrupted cells of *Tetradesmus obliquus* after high-pressure homogenization at different pressure or cycles, estimated from microscopic analyses. Examination of (**b**) optical and (**c**) fluorescence microscopy using *Tetradesmus obliquus* stained with SYTOX Green after high-pressure homogenization treatment using 350 bar/2 cycles. Red-coloured cells are due to chlorophyll fluorescence (intact cells); green-coloured cells are due to SYTOX Green staining (permeabilised cells).

Microscopy results revealed equal trends as FC analyses, with intact cells decreasing over increased pressure or number of cycles, accompanied by an increase in the permeabilised and disrupted cells population (Figure 4a). Although maximal cell disruption was similar to both analyses (75% for FC and 79% for microscopy, at 600 bar and 3 cycles), the distribution of the different cell populations was not as logical as FC analyses (namely at 100 bar/3 cycles and 600 bar/2 cycles). Still, optical (Figure 4b) and fluorescence (Figure 4c) microscopy is a simple method to analyse the impact of cell disruption treatments qualitatively, by allowing the visualization of cell debris and remaining cell populations. For precise quantitative analyses of disrupted cells, FC is more reliable than haemocytometer manual counts [33].

Cellular disruption and the applied HPH pressures are dependent on the microalgae cell wall composition, which varies greatly among species. For instance, while 170 bar was enough to disrupt 50% of *Tetraselmis* sp. Cells, 1380 and 2000 bar was needed to equally disrupt *Chlorella* sp. And *Nannochloropsis* sp., respectively [34]. As for *Tetradesmus* sp., a significant release of intracellular components as a result of high shear stress was only observed at pressures equal to or above 600 bar [26]. Distinguishing the level of cell destruction is then crucial to optimize HPH in microalgae-based processes.

### 2.2. Wheat Seed’s Germination

**Figure 5:**
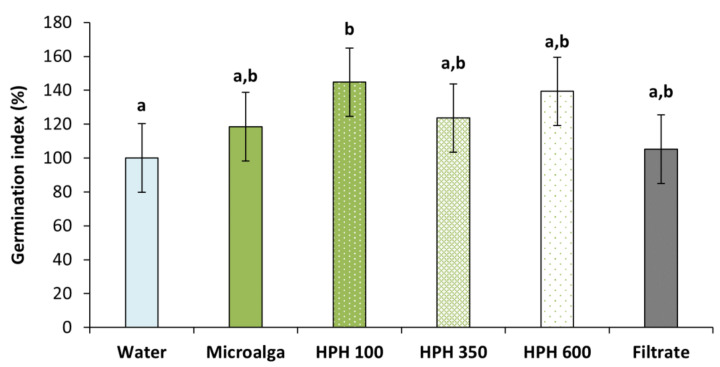
Germination index of wheat plants treated with *Tetradesmus obliquus* after high-pressure homogenization at 100, 350, and 600 bar (1 cycle) and non-disrupted microalga culture, and its filtrate, compared to distilled water (control). Different letters indicate significant differences among treatments (Tukey’s test, *p* < 0.05) and data are shown as mean ± pooled standard deviation (*n* = 3).

To assess the germination inducing capacity of *Tetradesmus obliquus*, the germination index (GI) was determined (Figure 5). All treatment conditions resulted in higher GI compared to distilled water (control). However, only the disrupted biomass at 100 bar was significant (*p* < 0.05), increasing 45% the GI, followed by 39% at 600 bar and 24% at 350 bar. These results suggested the need for a disruption method such as HPH. The non-disrupted microalga achieved an increase of almost 20%, while the filtrate (culture medium after filtration of the non-treated culture,) only had a 5% rise.

The pressure intensification of the treatment resulted in a decrease in wheat GI. Indeed, at 350 and 600 bar permeabilised and disrupted cells combined were higher than at 100 bar, leading to a greater release of intracellular components with biostimulant effect (Figure 2). This suggests that milder pressures are more beneficial than stronger ones, which could be due to an inhibitory effect resulting from too high biostimulant compound concentrations, as already reported previously by Navarro-López et al. (2020a) [20] and Ferreira et al. (2021) [23]. On the other hand, harsher HPH conditions could eventually lead in some extent to the increasing degradation of beneficial intracellular compounds, conducting to a less positive effect on biostimulation.

**Figure 6:**
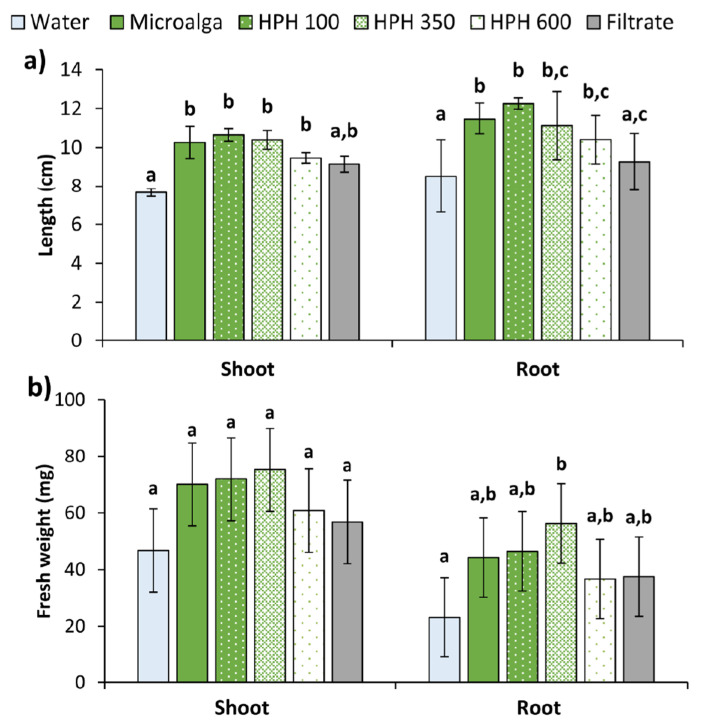
(**a**) Length, and (**b**) fresh weight of shoot and root of wheat plants treated with *Tetradesmus obliquus* after high-pressure homogenization at 100, 350, and 600 bar (1 cycle), non-disrupted microalga culture, and its filtrate compared to distilled water (control). Different letters indicate significant differences among treatments (Tukey’s test, *p* < 0.05) and data are shown as mean ± pooled standard deviation (*n* = 3).

In the Petri dishes control (distilled water), almost every seed germinated, though the development of the seedlings was reduced. Contrary, the non-disrupted microalga had a greater effect on the seedling growth in both shoot and root, as shown in Figure 6. Aside from the filtrate, all the treatments applied to the seeds produce significantly longer shoots and roots than the control (*p* < 0.05), but do not differ amongst themselves (Figure 6a). The filtrate generated statistically longer shoots than the control (*p* < 0.05), although lower compared to the other treatments. No differences were found in root length between the filtrate and the control (*p* > 0.05). All treatments also resulted in a higher fresh weight of plants compared to water. In shoots, an increase of 61% for 350 bar, followed by 54% for 100 bar, 50% for non-disrupted microalga, and 30% for 600 bar. In roots, the same trend was observed (144, 101, 92, and 59%) for the fresh weight. In addition, the filtrate allowed an increase of 22 and 62% in shoots and roots of treated plants, respectively. However, the increments in shoots were not significant (*p* > 0.05), while for roots only the 350 bar was significantly higher than the control (*p* < 0.05).

Based on all results, the non-disrupted culture, as well as the lowest pressure (100 bar) should be highlighted from the economical and energetical point of view, since they allow a similar increase in plant growth at lower processing requirements. On the other hand, although achieving lower increments in both shoots and roots growth of wheat plants, the filtrate should be further studied as a possibility of using the treated wastewater as supplemented irrigation water, since some microalgae can release bioactive compounds to the medium. In addition, the supernatant fraction obtained by centrifugation of the HPH disrupted biomass could also be interesting to study as it will most certainly be rich in growth-promoting molecules released from the cell. This could allow the use of the microalga biomass for agricultural and other applications, towards a zero-waste process.

The disruption of the cell wall seems to be crucial for the bioavailability of bio compounds, e.g., on juvenile fish Nile tilapia diet [35]. However, both Navarro-López et al. (2020a) [22] and Ferreira et al. (2021) [23] showed no need for disruption to obtain an increase of biostimulation effect, but used lower biomass concentrations (0.2 and 0.5 g/L). A later study of Navarro-López et al. (2020b) [26] reported increased watercress germination after *Scenedesmus* sp. being subject to 200 bar but no significant effects on higher pressures. More disrupted biomass leads to higher availability of bioactive compounds, which seems to hinder the germination index and growth of seedlings. However, it is known for a long time that HPH can result in the degradation of compounds, such as lowering protein digestibility [36]. Moreover, Stirk et al. (2020) [25] pointed out that both sonication and ball-milling hinder germination on mung beans. They claimed that bioactive compounds in microalgae are sensitive to post-harvest processes and their biological activity can be negatively affected by cell disruption methods. Although the impact of HPH on phytohormones viability has not been assessed so far, high shear forces could be compromising their bioactivity. Indeed, decreased contents of carotenoids were found by subjecting samples to 1000 bar [37], or decreased contents of vitamin C and anthocyanin after 2 cycles at 500 bar [38].

## 3. Materials and Methods

### 3.1. Effluent and Microalga

The piggery wastewater (PWW) was collected from a stabilization pond in a local pig farm from Valorgado in Herdade do Pessegueiro (39°00′09.0″ N, 8°38′45.5″ W) (Glória do Ribatejo, Portugal). This effluent corresponds to the liquid fraction of pig slurry after separation from solid manure. The effluent composition is shown in Table 1. The microalgae selected for this work was *Tetradesmus obliquus* (formerly known as *Scenedesmus obliquus*) (ACOI 204/07, ACOI Culture Collection, Coimbra University, Portugal), after a screening done with several other species (Ferreira et al., 2021 [23]). The microalga was cultivated in piggery wastewater diluted 1:20 in a 5 L bubble-column photobioreactor with a working volume of 4 L. The culture was kept at room temperature (23–25 °C), under continuous artificial light using fluorescent lamps (Philips TL-D) at 67.6 μE/(m^2^·s), supplied with an air flow rate of 0.7 vvm. The culture was collected after 16 days (at stationary phase), with a concentration of 0.8 g/L (ash-free dry weight), for the further disruption tests as well as for the direct use as a biostimulant in seed germination and plant growth.

### 3.2. Cell Disruption by High-Pressure Homogenizer (HPH)

*T. obliquus* culture grown in PWW was submitted to different high-pressure homogenization (HPH) conditions of pressure and number of cycles to test the influence of these factors on cell integrity for releasing and extracting compounds with agricultural interest. Cell disruption was carried out in a PandaPLUS 2000 homogenizer (GEA, Düsseldorf, Germany) by passing the microalga culture, at a concentration of 0.8 g/L (ash-free dry weight), under different pressures (100, 350 and 600 bar) and cycles (1, 2 and 3). Samples were collected and immediately directed to FC analysis to evaluate cell integrity.

### 3.3. Flow Cytometry for Evaluating Cell Disruption

The disrupted microalga samples were analysed in a CytoFLEX Flow cytometer (Beckman Coulter Life Sciences, Brea, CA, USA), equipped with a 488 nm argon laser. Fluorescence data was collected in the Forward (FSC) and Side Scatter (SSC) detectors, as well as the FITC (green) fluorescence detector. FC distinguishes cells with different sizes and internal complexities, based on their light scatter properties, which are detected in FSC and SSC detectors. For *T. obliquus* cell membrane integrity detection, samples were stained with the viability dye SYTOX Green (1.8 µM, for 25 min, in the dark). Before analysing HPH treated *T. obliquus* cells, a set of controls was previously carried out to verify if SYTOX Green could detect *T. obliquus* cells with damaged membranes. The positive control used heat-treated *T. obliquus* cells (incubated in a water bath at 100 °C for 60 min) to induce massive cell membrane permeabilisation, before staining with SYTOX Green. Microalgae samples were diluted with phosphate saline buffer (PBS) to adjust cell concentration to 100–150 events per second.

This methodology distinguishes the following *T. obliquus* cell sub-populations:

(i) Non-stained cells (SYTOX −) with intact cell membrane (intact cells), located in the lower quadrant of the FITC/SSC density plot;
(ii) Stained cells (SYTOX +) located in the upper quadrant of the FITC/SSC density plot, which, despite maintaining their size and internal content (since they were in the same FSC/SSC gate as untreated cells) had permeabilised membrane (permeabilised cells);
(iii) Cell debris caused by severe disruption, located at lower FSC and SSC ranges (disrupted cells).

Percentages of these populations were calculated according to the following equations:

(1) $$Intact\;cells\;\left(\%\right)=\frac{Non\;stained\;treated\;cells\;\left(Sytox\;-\right)}{Total\;untreated\;cells}$$

(2) $$Permeabilized\;cells\;\left(\%\right)=\frac{Stained\;treated\;cells\;\left(Sytox\;+\right)}{Total\;untreated\;cells\;}\;$$

(3) $$Disrupted\;cells\;\left(\%\right)=\frac{Total\;untreated\;cells-Total\;treated\;cells}{Total\;untreated\;cells}\;$$

### 3.4. Fluorescence Microscopy for Evaluating Cell Disruption

Microalgae cells after HPH were also examined using fluorescence microscopy. *T. obliquus* samples were incubated at room temperature with 1.8 μM SYTOX for 25 min and in the dark. 10 μL of the mixture was added to a Neubauer hematocytometer (0.1 mm depth) and cells were observed using a LEICA DLMB (Leica Camera AG, Germany) equipped with an HBO-100 mercury lamp and filter set of UV, blue and green filters. Neabauer manual counts were done according to Vega and Voltolina (2007) [40], and cell populations were calculated using FC equations (Equations (1)–(3)). Intact cells were spotted by their chlorophyll fluorescence (red; SYTOX −), while permeabilised cells were identified by their SYTOX fluorescence (green; SYTOX +). Cells that maintained their size and internal structure (total treated or untreated ce lls) were visualized by optical microscopy.

### 3.5. Germination Test on Wheat Seeds

The biostimulant effect of *T. obliquus* culture was determined in germination bioassays using wheat seeds (*Triticum aestivum*). For each condition, 15 seeds were placed in Whatman filter paper, in three Petri dishes, and then treated with 10 mL of distilled water (control), culture without any treatment, and the homogenized cultures (after 100, 350, and 600 bar for 1 cycle), as well as the culture’s filtrate (Whatman GF/C filter). The latter corresponds to the culture medium (piggery wastewater after microalga growth) after filtration, meaning without the microalga cells that were retained in the filter. This condition was done to evaluate the possibility of the cells releasing some extracellular compounds with biostimulant effect, with the advantage of using both fractions.

Minor dilutions of the culture can occur when operating the HPH due to necessity of operating in wet conditions (adding water before and after passing the culture). Thus, the final concentration (AFDW) of the processed culture is presented in Table 2.

The dishes were maintained at room temperature (around 25 °C) for 5 days, and the seeds were watered daily with the same amount of distilled water to keep the filter paper humid.

In the end, the seedlings were carefully separated and measured with a ruler. Then, the plants were divided into shoots and roots to determine their fresh weight.

Finally, the germination index (GI) of each sample was determined by Equation (4) according to Zucconi et al. (1981) [41]:

(4) $$GI\;\left(\%\right)=\frac{G\times L}{G_{w}\times L_{w}}\times 100\;$$

where *G* and *L* are the number of germinated seeds and the root length for microalgae extracts and *G_w_* and *L_w_* are the same parameters for the control (distilled water).

### 3.6. Statistical Analysis

One-way ANOVA was used to describe the effects the microalga at different conditions on the germination index and growth of wheat seedlings. The *p*-values resulting from the sum of square analyses were used to describe the impact of the factors, while Tukey’s post hoc test was used to detect differences among treatments. For all tests a significance level (*α*) of 0.05 was considered and outliers were disregarded.

## 4. Conclusions

Algae offer great potential to support the building of a bio-based economy and can contribute to new solutions to overcome some of the big challenges of the twenty-first century. This work focused on wastewater treatment with the recovery of the nutrients and the valorisation of all the fractions as biostimulant and enriched irrigation water for wheat plants in a circular economy. The FC coupled with SYTOX Green was confirmed to be a robust and fast tool to analyse *Tetradesmus obliquus* cellular integrity after being subjected to disruption with a high-pressure homogenizer. It was possible to distinguish between intact, permeabilised and disrupted cells and correlate *T. obliquus* effects in wheat plants. Cellular disruption by HPH appeared to promote the bioavailability of the microalga compounds by increasing seed germination and plant growth at 100 bar. This method could be extended to other agricultural applications as well as for food and feed industries, therefore enabling faster development of microalgae-based products. Nonetheless, optimization studies of HPH procedure will be necessary since it is dependent on the microalga species, as well as cultivation medium and state of culture. Moreover, the final application of the biomass should also be considered, since the optimal conditions might differ. Finally, future studies could test the concentration of bioactive compounds and evaluate the effect of the fractions obtained after centrifugation of the HPH biomass: the concentrate comprising the whole biomass that could be targeted to food or feed, and the supernatant containing the bioactive compounds released from the microalgal cells could be applied to agriculture, establishing a biorefinery to take advantage of all the biomass potential.

## Figures and Tables

**Table 1:** Composition of the piggery wastewater (PWW) and diluted 1:20, in terms of chemical oxygen demand (COD), ammonia nitrogen (NH_4_^+^), phosphate (PO_4_^3−^), total suspended solids (TSS), and volatile suspended solids (VSS) [39].

Effluent	COD (mg O_2_/L)	NH_4_^+^ (mg/L)	PO_4_^3−^ (mg/L)	TSS (mg/L)	VSS (mg/L)
PWW	3759 ± 71	1500 ± 7	97.5	2575 ± 45	1780 ± 20
1:20 PWW	184.4 ± 71	64.6 ± 1.1	6.40	90.0 ± 0.00	72.5 ± 2.5

**Table 2:** *Tetradesmus obliquus* concentration in ash-free dry weight (AFDW) of the different treatments tested for wheat germination.

Condition	AFDW (g/L)
Non-treated	0.80
100 bar	0.84
350 bar	0.79
600 bar	0.74

## Data Availability

The data presented in this study are available on request from the corresponding author.
